# Is glaucoma blindness a disease of deprivation and ignorance? A case–control study for late presentation of glaucoma in India

**DOI:** 10.4103/0301-4738.73720

**Published:** 2011

**Authors:** Parikshit Gogate, Roma Deshpande, Vidya Chelerkar, Swapna Deshpande, Madan Deshpande

**Affiliations:** HV Desai Eye Hospital, Pune, Maharashtra, India; 1 Consultant Biostatician, Pune, Maharashtra, India

**Keywords:** Awareness, blindness, deprivation, glaucoma, late presentation

## Abstract

**Aim:**

The aim was to identify the presenting symptoms and social risk factors for late presentation of primary glaucoma in newly diagnosed cases.

**Materials and Methods:**

It was a case-control study in a tertiary eye care center in Maharashtra, India. Newly diagnosed patients with primary glaucoma were classified as cases (late presenters) where there was no perception of light in one eye or severe visual field loss affecting an area within 20° of fixation or a cup–disc (C:D) ratio ≥0.8 and controls (early presenters), presenting relative scotoma within 20° of fixation or a C:D ratio <0.8, but >0.5. All patients underwent a comprehensive ocular examination including gonioscopy, perimetry, and detailed family and social history. Occupation, education, and socioeconomic status were graded. SPSS version 12.0 was used, and univariate and multivariate logistic regression analysis was performed.

**Results:**

Gradual progressive painless loss of vision was the commonest symptom (175, 87.5%). Primary angle closure glaucoma was more common in females (*P* = 0.001) and lower socioeconomic groups (*P* = 0.05). Patients who were less educated were more likely to have late presentation of glaucoma (*P* < 0.001, odds ratio = 0.07; 95% CI, 0.02–0.25). Knowledge of family history of glaucoma (*P* = 0.80, odds ratio = 1.16; 95% CI, 0.36–3.71) and eye clinic attendance in past 2 years still resulted in late presentation (*P* = 0.45, odds ratio = 1.34, 95% CI, 0.63–2.82).

**Conclusion:**

Lack of education and awareness of glaucoma were major risk factors for late presentation.

Glaucoma is one of the leading causes of blindness in most parts of the world.[1–8] Despite new medical and surgical strategies to control intraocular pressure (IOP), blindness caused by glaucoma continues to increase, and glaucoma remains the second or the third most common cause of blindness in the world.[9,10] As glaucoma is a disease with few symptoms in initials stages, late presentation is common and, when visual field loss threatens central vision, is an important risk factor for blindness.[11,12]

While there have been few studies demonstrating the association of late presentation of glaucoma with social factors from the UK,[13,14] there has been none from India where glaucoma is a significant cause of blindness.[7] Lack of awareness about glaucoma also contributes to its late presentation. In the Barbados Eye Study (BES), about half of the total number of persons with prevalent primary open angle glaucoma (POAG, 51%) were unaware of their diagnosis.[15] Some communities in developed countries like the UK too had lack of awareness.[16] The Andhra Pradesh Eye Disease Study (APEDS) showed that awareness of glaucoma was very limited in the rural areas of southern India.[17] To our knowledge no such study has been conducted in Maharashtra in western India.

We undertook a case–control study to determine the association between social factors, awareness, and late presentation of glaucoma in a tertiary eye care center in Maharashtra, West India.

## Materials and Methods

A hospital-based case–control study was conducted involving patients diagnosed with primary glaucoma for the first time at visit to a tertiary eye care center in Pune, Maharashtra, India.

Newly diagnosed patients with primary glaucoma were selected. A complete ophthalmic examination was performed including best corrected visual acuity (BCVA), IOP measurement by applanation tonometer and fundus examination for the cup:disc ratio (C:D ratio), gonioscopy by Goldman’s three-mirror goniolens, and visual field test by the Humphrey automated perimeter (30-2 glaucoma threshold). The Hodapp–Parrish–Anderson visual field grading scale was used for the field defects. Glaucoma was diagnosed if the C:D ratio was >0.5 or if there was a difference of >0.2 between the two eyes with corresponding field defects. POAG and primary angle closure glaucoma (PACG) were differentiated by gonioscopy.

Patients were classified as early and late presenters using the following criteria: early presenters (controls) had visual field with no absolute scotoma within 20° of fixation or C:D ratio >0.5 and <0.8 or a difference of >0.2 between the two eyes. Late presenters (cases) had no perception of light or severe visual field loss affecting an area within 20° of fixation or a C:D ratio > 0.8. Patients with secondary glaucoma, congenital and juvenile glaucoma, previous history of glaucoma, suspected glaucoma, and other optic nerve pathologies were excluded from the study.

The study was approved by the hospital’s ethical committee. Informed consent of the patient was taken as per Helsinki guidelines and the patient was presented with a questionnaire to get information regarding symptoms, education, occupation, travel time, travel expenses, occupation and education of the head of the family, type of housing, number of household members, per capita income, earning status, family history of glaucoma, degree of relationship with the head of the family, visits to ophthalmologists in the past 2 years, difficulty in navigation, affordability of treatment, awareness about glaucoma, and willingness for compliance.

Travel time was the time required to travel from home to the hospital and was noted in hours. Travel expenses required to reach hospital were noted in rupees ($1= Rs 46 by 2007 exchange rate). These were indirect and direct proxy of cost for the patients to reach the hospital. Occupation, education, and socioeconomic status were graded according to Prasad’s classification for rural population [Table 1],[18] and Kuppuswami’s classification for urban population [Table 2].[19] The patients were asked to report their level of education and their occupation. The type of housing was investigated, *kaccha* (without use of cement, mud huts, or shacks) or *pucca* (with use of bricks and cement).

**Table 1:** Prasad’s method of social classification (rural)

Social class	Per capita income per month (INR)
I	>2504
II	1253–2503
III	277–1252
IV	250–276
V	<250

**Table 2:** Kuppusamy’s method of social classification (urban)

Item		Score
A. Education		
Professional degree, honors degree, postgraduate degree		7
Graduation		6
Intermediate, post-high-school diploma		5
High school certificate		4
Middle school certificate		3
Primary school or literate		2
Illiterate		1
B. Occupation		
Professional		10
Semiprofessional		6
Clerk, shop owner, farm owner		5
Skilled worker		4
Semiskilled worker		3
Unskilled worker		2
Unemployed		1
C. Per capita income per month		
≥2000		12
1000–1999		10
750–999		6
500–749		4
300–499		3
101–299		2
≤100		1
Calculation:		
Total score = (A + B + C)	Social class	
26–29	I	
16–25	II	
11–15	III	
05–10	IV	
<5	V	

Awareness of glaucoma was determined by asking the patient if he/she knew or had heard about glaucoma. Furthermore, the patient was asked if he/she was aware about the role of intraocular pressure, the possibility of visual field loss and optic nerve damage, the progressive nature of the disease, its irreversible nature, the meticulous need for compliance of treatment, and predisposition due to family history. Social risk factors of glaucoma were studied by comparing the association of various socioeconomic factors with early and late presentation of glaucoma. The patients were not aware of their case or control status. A pilot study was conducted first. The data collection form is enclosed as Appendix A.

One hundred glaucoma patients in each group of early and late presenters were enrolled to detect if there was a 10% difference, between the lower and higher socioeconomic class at 80% power with a 95% confidence level.

Data were presented with mean (standard deviation, SD) or number (%) in the case of continuous or categorical data, respectively. In the statistical analysis, variables with skewed distribution were log-transformed to satisfy the assumption of normality. For continuous variables, the mean difference between groups was tested using the independent t-test, and using the chi-square test or Fisher exact test in the case of categorical data. Risk factors for determinants of late presentation of glaucoma were tested using univariate and multivariate logistic regression analysis. Two-sided P-value less than 0.05 was considered significant. Statistical Package for Social Sciences (SPSS) version 12.0 for windows (SPSS Inc., Chicago, IL, USA) was used for statistical analysis.

## Results

The first hundred early and late presenters between June 2006 and December 2007 were included in the study. The patients were from Pune and Solapur districts of Western Maharashtra. One hundred and fifty-one were POAG cases and 49 were with PACG. Except two, all the PACG cases were chronic angle closure glaucoma. A total of 97 out of 151 (64.2%) patients with POAG and 17 out of 49 (34.7%) with PACG were males. Fourteen (7%) were less than 40 years old, 92 (46%) were 41–60 years of age, and 93(46.5%) were 61–80 years of age. Only one patient was more than 80 years old. The average age of early presenters was 59.5 years (SD 12.4) and of late presenters was 59.4 years (SD 10.5). Fifty-three early and 33 late presenters were females (*P* < 0.001). The level of education among early presenters was illiterate and primary school 15%, middle and high school 35% and graduate, postgraduate, and diploma 50%; for late presenters it was 42%, 37%, and 21%, respectively (*P* < 0.001). The occupations of early presenters were unemployed and unskilled workers 12%, semiskilled and skilled workers 54%, semiprofessionals and professionals 34%; for late presenters, it was 32%, 42%, and 26%, respectively (*P* = 0.003).

Painless diminution of vision was the most common symptom in the patients (*n* = 175, 82.5%) as shown in Table 3.

**Table 3:** Presenting symptoms

Symptom	POAG	PACG	Total
Painless DOV	134	41	175
Painful DOV	16	22	38
Headache	8	17	25
Redness	6	9	15
Colored halos	12	7	19
Difficulty in near work	5	0	5
Difficulty in night vision	4	0	4
Difficulty in navigation	15	0	15
Routine check-up	7	0	7
Other	28	0	28

DOV: Diminution of vision, POAG: Primary open-angle glaucoma, PACG: Primary angle closure glaucoma

PACG cases presented with redness, colored halos, and headache. Colored halos were also present in POAG cases; this may be attributed to lenticular opacity in these cases. Most patients presented with more than one symptom.

Table 4 shows the social risk factors for late presentation of glaucoma. Travel time and expense were significantly related to each other (*P* < 0.001). Eighteen early and 31 late presenters spent >3 h to reach the hospital. Increased travel time (i.e., time required to travel from home to hospital) was not statistically related to the late presentation of glaucoma. Only 16% among early and 28% among late presenters spent >Rs 100 in reaching the clinic. Patients who had to spend more money to reach the hospital were late presenters compared to patients who had to spend less money (*P* = 0.043, odds ratio 2.04, 95% CI, 1.02–4.07); but this was not significant by multivariate analysis.

**Table 4:** Analysis of social risk factors of late presentation of glaucoma

	Univariate models		Multivariate model	
	Odds ratio	*P*-value	AOR	*P*-value
Sex				
Female	1		1	
Male	2.28 (1.29, 4.05)	0.005	3.31 (1.50, 7.31)	0.003
Age group				
<60 years	1		1	
≥60 years	1.34 (0.76, 2.35)	0.32	1.06 (0.53, 2.12)	0.86
Travel time (h)				
<1	1		1	
1–3	0.59 (0.31, 1.14)	0.117	0.45 (0.21, 0.99)	0.047
≥3	1.52 (0.71, 3.25)	0.28	0.56 (0.17, 1.78)	0.324
Traveling expenses (INR)				
<100	1		1	
≥100	2.04 (1.02, 4.07)	0.043	1.58 (0.58, 4.29)	0.367
Occupation				
Unemployed and unskilled workers	1		1	
Semiskilled and skilled workers	0.29 (0.13, 0.63)	0.002	0.40 (0.16, 1.02)	0.055
Semiprofessional and professionals	0.29 (0.12, 0.66)	0.003	0.67 (0.19, 2.41)	0.55
Education				
Illiterate and primary schooling	1		1	
Middle school and high school	0.38 (0.18, 0.80)	0.011	0.34 (0.14, 0.82)	0.018
Graduate, PG, and diploma	0.15 (0.07, 0.32)	0.001	0.07 (0.02, 0.25)	< 0.001
Housing				
Pucca	1		1	
Kaccha	1.96 (1.10, 3.49)	0.022	0.98 (0.42, 2.31)	0.98
Income/month (INR)				
≤1000	1		1	
≥1000	1.13 (0.64, 2.00)	0.67	0.49 (0.20, 1.22)	0.127
Head of the family	1		1	
Other members in the family	0.65 (0.36, 1.16)	0.142	1.23 (0.54, 2.79)	0.611
Family history of glaucoma present	1		1	
Family history of glaucoma absent	0.80 (0.32, 2.02)	0.64	1.16 (0.36, 3.71)	0.80
Attended eye clinic	1		1	
Not attended eye clinic	1.88 (0.99, 3.59)	0.055	1.34 (0.63, 2.82)	0.458

AOR: Adjusted odds ratio, INR: Indian rupees, PG: Postgraduate

Occupation was a risk factor for late presentation of glaucoma. Patients who were semiskilled or skilled workers were less likely to present late as compared to unemployed or unskilled workers (*P* = 0.002, odds ratio 0.29, 95% CI, 0.13–0.63). Patients who were doing semiprofessional and professional work were also less likely to be late presenters (*P* = 0.003, odds ratio 0.29, 95% CI, 0.12–0.66). But this was not significant after multivariate analysis. Poor education was an independent correlate of late presentation of glaucoma (*P* < 0.001, odds ratio 0.07, 95% CI, 0.02–0.25). It remained significant even after adjusting for other factors in multivariate analysis.

Family status was not a risk factor for late presentation of glaucoma in Maharashtra; relationship with the head of the family, whether head or other dependent member of the family, did not affect the stage of presentation of glaucoma (*P* = 0.611).

The type of housing, which is one of the indicators of the socioeconomic status, did affect the stage of presentation of glaucoma (*P* = 0.022, odds ratio 1.96, 95% CI, 1.10–3.49). People living in *kaccha* houses (mud huts or shacks) were likely to present late. This was not significant by multivariate analysis.

Twenty (10%) patients had a positive family history of glaucoma, 16 had POAG, and 4 had PACG. Eleven out of 20 (55%) were early presenters and 9 of 20 (45%) were late presenters. Family history of glaucoma was a mild preventative factor for late presentation of glaucoma (*P* = 0.80, odds ratio 1.16, 95% CI, 0.36–3.71).

Frequenting an eye clinic for 2 years was not found to be preventative for late presentation of glaucoma (*P* = 0.45, odds ratio 1.34, 95% CI, 0.63–2.82). In 26 patients, glaucoma was not diagnosed in the last 2 years by ophthalmologists (6 patients) or optometrists (20 patients). Sixteen of these were late presenters.

Among the POAG patients, 47.7% were early presenters and 52.3% were late presenters. Among PACG patients, 57% were early presenters and 43% were late presenters. This study showed that professionals commonly presented higher incidence of POAG and lower incidence of PACG, as compared to unemployed and lower occupational group patients (*P* = 0.05), as shown in Table 5. Females were more likely to have PACG (*P* = 0.001) than POAG.

**Table 5:** Analysis of the presenting pattern and type of glaucoma

	POAG *N* = 151	PACG *N* = 49	*P*-value
Age (mean, SD), years	59.9 (10.9)	58.2 (13.0)	0.369
	*n* (%)	*n* (%)	
Sex			
Male	97 (64.2)	17 (34.7)	0.001
Female	54 (35.8)	32 (65.3)	
Occupation			
Unemployed and unskilled workers	30 (19.9)	14 (28.6)	0.103
Semiskilled and skilled workers	70 (46.4)	26 (53.1)	
Semiprofessionals and professionals	51 (33.8)	9 (18.4)	
Education			
Illiterate and primary schooling	43 (28.5)	14 (28.6)	0.42
Middle school and high school	51 (33.8)	21 (42.9)	
Graduate, —PG, and diploma	57 (37.7)	14 (28.6)	
Socioeconomic status			
I	29 (19.2)	3 (6.1)	0.05
II	46 (30.5)	14 (28.6)	
III	39 (25.8)	13 (26.5))	
IV	24 (15.9)	16 (32.7)	
V	13 (8.6)	3 (6.1)	
Family history			
Yes	16 (10.6)	4 (8.2)	0.826
No	135 (89.4)	45 (91.8)	

POAG: Primary open-angle glaucoma, PACG: Primary angle closure glaucoma

Table 6 shows the relationship of social factors with awareness about glaucoma.

**Table 6:** Analysis of social factors and awareness of glaucoma

	Aware of glaucoma *N* = 17	Unaware of glaucoma *N* = 183	*P*-value
Age mean (SD), years	53.0 (12.8)	60.0 (11.12)	0.015
Sex	*n* (%)	*n* (%)	
Male	9 (52.9)	105 (57.4)	0.92
Female	8 (47.1)	78 (42.6)	
Occupation			
Unemployed and unskilled workers	5 (29.4)	39 (21.3)	0.276
Semiskilled and skilled workers	5 (29.4)	91 (49.7)	
Semiprofessionals and professionals	7 (41.2)	53 (29.0)	
Education			
Illiterate and primary schooling	3 (17.6)	54 (29.5)	0.58
Middle school and high school	7 (41.2)	65 (35.5)	
Graduate, PG, and diploma	7 (41.2)	64 (35.0)	
Socioeconomic status			
I	3 (17.6)	29 (15.8)	0.847
II	5 (29.4)	55 (30.1)	
III	6 (35.3)	46 (25.1)	
IV	2 (11.8)	38 (20.8)	
V	1 (5.9)	15 (8.2)	
Relation with head of the family			
Head	9 (52.9)	63 (34.4)	0.21
Others	8 (47.1)	120 (65.6)	
Family history			
Present	8 (47.1)	12 (6.6)	<0.001
Absent	9 (52.9)	171 (93.4)	

Only 17 (8.5%) patients were aware of glaucoma. Those who were aware of glaucoma were younger as compared to those unaware (*P* = 0.015). Awareness of glaucoma was higher among those having a positive family history (*P* < 0.001). Eight out of 20 (40%) patients with a positive family history had incomplete information about glaucoma. Awareness was poor among both genders; family members and occupational classes and grade of occupation did not affect awareness of glaucoma (*P* = 0.276). Awareness of glaucoma was poor in all uneducated class patients, but was slightly higher in educated patients (9.8%) than in illiterate patients (5.3%); but this was statistically insignificant (*P* = 0.58).

## Discussion

Gradual painless diminishing vision was the most common symptom for presentation. Glaucoma was an incidental finding in the majority of cases in our study. This proves the importance of comprehensive eye examination in every patient who attends an eye clinic.

In higher socioeconomic status patients, the prevalence of POAG was higher and that of PACG was lower when compared to those with a lower socioeconomic status (*P* = 0.05). Further studies are needed to corroborate these findings. Similar findings were observed in the APEDS from Andhra Pradesh.[20] PACG was more common in women than men. This corroborated the findings of two population-based studies from India, the APEDS[20] and the Chennai Glaucoma Study.[21]

Socioeconomic status was a risk factor for late presentation of glaucoma. A recent study from Scotland, UK, showed that areas with higher index of deprivation had more severe glaucoma on presentation to the health system.[22]

Glaucoma tends to run in families.[4,23–25] A study from Boston showed that people who had a first-degree relative with glaucoma were more aware of the disease.[26] In Moorefield’s Eye Hospital Study, it was observed that stronger the patient’s family history, the lower the odds of late attendance.[13,14] But in our study, even patients with a positive family history were likely to present late as awareness of glaucoma was very poor. Perhaps the ophthalmologists who treated the affected family member did not take time to counsel the patient that the disease runs in families and all siblings and children should undergo a regular comprehensive eye examination.

It was observed that in some cases, the glaucoma diagnosis was missed by optometrists and ophthalmologists, perhaps because a comprehensive eye examination was not performed. Similar findings were observed in the Barbados Eye Studies where visits to the optometrists still left many patients unaware about their glaucomatous condition.[15] Optometrists and ophthalmic assistants should also be educated about glaucoma as they reach a large sector of the population, in rural areas, which does not have access to a comprehensive eye care center.

In our study, awareness about glaucoma was very poor across all categories of patients. According to APEDS, those who were illiterate and from a poor socioeconomic class in rural India were less aware of glaucoma,[17] as in the Chennai Glaucoma Study.[27] Similar trend was reported from the BES from Barbados, USA, and Australia.[15,26,28,29] In our study, it was observed that the younger group was more aware of glaucoma than the older group, similar to a US study.[26] Even patients with a family history of glaucoma were not fully aware about the irreversible nature and need for regular treatment and follow-up, unlike those from Nigeria[30] and USA.[26]

An Australian study showed that the lack of awareness of glaucoma was a major risk for late presentation, rather than the lack of access to care.[29] Improving education and increasing awareness of glaucoma can go a long way in decreasing the late presentation of the disease. Information about the progressive and irreversible nature of disease, the need for meticulous compliance, predisposition due to a positive family history, and importance of a regular follow-up should be given to all glaucoma patients and suspects. Information brochures and pamphlets should be given to all out-patients visiting an eye clinic and posters with glaucoma information displayed. Mass media like radio, television, and newspapers should also be utilized to increase awareness of glaucoma, as was done in Ealing, UK.[31] The public education campaign should not however raise unnecessary alarm about the disease, and should encourage only those at risk to seek treatment, rather than indiscriminately increasing the workload of eye clinics, which would be counter-productive and may be misused.

This is a case–control study from a single center and thus has its limitations. In univariate logistic regression, factors like gender, education, occupation, housing type, and traveling expenses to reach the hospital facility were significant correlates of late presentation. These were unadjusted relations between covariates and outcome variables. Gender and education remained as independent significant predictors of late presentation after adjusting for all other cofactors in the multivariate logistic regression model. Also there was lack of awareness of glaucoma among all subgroups and some cases were missed on previous examination in a different clinic.

Lack of education and awareness of glaucoma among patients were the major risk factors for late presentation. A comprehensive eye examination should be done for every patient attending an eye clinic and this should be stressed in ophthalmologists’ and optometrists’ training programs as this was the best method to diagnose glaucoma.

## Appendix A

**Table:**

Data collection form			
Name:	Age/sex:		
Address:	Registration number:		
Date:			
Travel time:	Travel expenses:		
Symptoms:			
Painless DOV- yes/no Painful DOV - yes/no DOV for near- yes/no Pain- yes/no Headache- yes/no Redness- yes/no Colored halos- yes/no None Others-			
Duration of symptoms-			
Examination:			

		**Right eye**	**Left eye**

Best corrected visual acuity			
Intra-ocular pressure			
C:D ratio			
Gonioscopy			
Visual field loss			
Anterior segment			
Posterior segment			

Diagnosis:			
Treatment:			
Place of residence: Urban/rural			
Social history:			
Occupation of head of family- Education of head of family- Housing- kaccha/pucca Number of people in the household- Per capita income Socioeconomic grade (as per Kuppusamy/Prasad classification)- Status in family- earning/nonearning Relation to head of family- Difficulty in navigation- yes/no Affordability of treatment- yes/no Knowledge about glaucoma- what is it? Do you know the importance of good compliance? Are you aware of its relationship with family history? Role of intraocular pressure? What is field loss? Is glaucoma blindness treatable?			
Compliance of treatment: yes/no			
Family history of glaucoma: yes/no			
Attended eye clinic/eye check-up in past 2 years: yes/no			
Check-up was done by: ophthalmologist/optometrist			
The presentation of glaucoma: early/late			
Diagram for cup disc ratio:			
